# Short-term atrial fibrillation onset prediction using machine learning

**DOI:** 10.1093/ehjdh/ztaf104

**Published:** 2025-09-11

**Authors:** Jean-Marie Grégoire, Cédric Gilon, François Marelli, Hugues Bersini, Laurent Groben, Thomas Nguyen, Bernard Deruyter, Pascal Godart, Stéphane Carlier

**Affiliations:** Cardiology Department, Université de Mons, Avenue Maistriau , 25, Mons 7000, Belgium; IRIDIA, Université Libre de Bruxelles, Av. Adolphe Buyl, Bruxelles 1000, Belgium; IRIDIA, Université Libre de Bruxelles, Av. Adolphe Buyl, Bruxelles 1000, Belgium; ISIA Lab, Université de Mons, Boulevard Dolez , 31, Mons 7000, Belgium; IRIDIA, Université Libre de Bruxelles, Av. Adolphe Buyl, Bruxelles 1000, Belgium; Cardiology Department, Centre Hospitalier de Luxembourg, Rue Nicolas Ernest Barblé, 4, Luxembourg 1210, G-D de Luxembourg; Cardiology Department, CHU Brugmann, Place A Van Gehuchten, 4, Bruxelles 1020, Belgium; Cardiology Department, Europe Hospitals, Av. De Fré 206, Bruxelles 1180, Belgium; Cardiology Department, CHU Helora, Bd John Fitzgerald Kennedy 2, Mons 7000, Belgium; Cardiology Department, Université de Mons, Avenue Maistriau , 25, Mons 7000, Belgium; Cardiology Department, CHU Helora, Bd John Fitzgerald Kennedy 2, Mons 7000, Belgium

**Keywords:** Machine learning, Deep learning, Atrial fibrillation, Prediction, Identification, Autonomic nervous system, Heart rate variability

## Abstract

**Introduction:**

Integrating machine learning (ML) models into wearable or connected devices to deliver early warning alerts prior to atrial fibrillation (AF) onset may represent an effective preventive strategy. Machine learning algorithms applied to two-lead Holter electrocardiogram (ECG) recordings can support the development of predictive models capable of detecting imminent paroxysmal AF episodes within short-term windows. This approach could facilitate a more targeted ‘pill-in-the-pocket’ (PITP)-like intervention strategy, potentially enhancing timely therapeutic actions and improving patient outcomes.

**Aim:**

This study aimed to identify patients currently in sinus rhythm who will experience an AF episode within the subsequent hours by analysing 24-h Holter ECG recordings with ML.

**Methods:**

We established a novel database comprising 95 871 manually analysed Holter ECG recordings, identifying 1319 episodes of paroxysmal AF from 872 patients. Among these, 835 AF episodes from 506 recordings had more than 60 min of normal sinus rhythm prior to AF onset and more than 10 min of sustained AF following onset. Patients were stratified into five age groups: all patients combined, under 60 years, 60–70 years, 70–80 years, and over 80 years. Additionally, 365 recordings from 347 patients without rhythm abnormalities were identified and classified, from which two ECG segments were selected. Two deep learning (DL) models were trained on raw ECG data to predict AF onset. To compare DL models with traditional ML approaches using heart rate variability (HRV) parameters, we employed a random forest classifier and a gradient-boosted decision tree model (XGBoost, XGB).

**Results:**

The decision trees models trained on HRV parameters delivered the best predictive performance. The most significant results were observed for episodes lasting more than 5 min of AF, achieving an area under the receiver operating characteristic curve of 0.919 (95% CI: 0.879–0.958) and an area under the precision–recall curve of 0.919 (95% CI: 0.879–0.958) for XGB. At a decision threshold of 0.5, accuracy was 84.5% (81.2–87.8), sensitivity was 83.0% (79.5–86.4), specificity was 86.6% (79.3–93.9), positive predictive value was 90.2% (85.5–94.9), negative predictive value was 78.4% (74.7–82.1), and the F1 score was 86.2% (83.5–89.0).

**Conclusion:**

These findings indicate that HRV parameters contain crucial information for the short-term prediction of AF onset, supporting preventive strategies. Integration of such predictive models into wearable mHealth technologies could facilitate a PITP-like preventive approach, potentially reducing AF-related morbidity. Prospective studies are warranted to validate these promising results further.

## Graphical Abstract

**Graphical Abstract:**
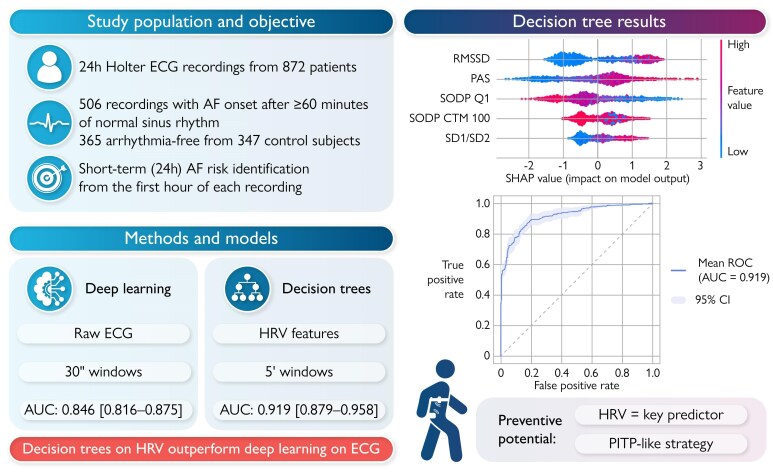
We analysed 24-h Holter ECG recordings from 872 patients with paroxysmal atrial fibrillation (AF) to assess short-term AF risk using only the first hour of data in normal sinus rhythm (NSR). As controls, 24-h recordings from 347 patients with NSR were included. We compared four models: two deep learning approaches trained on raw ECG signals and two decision tree models based on heart rate variability (HRV) features. The HRV-based models outperformed deep learning, with the best decision tree model achieving an area under the receiver operating characteristic (ROC) curve (AUC) of 0.919 [0.879–0.958], compared with 0.846 [0.816–0.875] for the top-performing deep learning model. SHapley Additive exPlanations analysis revealed five key HRV features driving the predictions. These findings support the potential of early warning alerts and pill-in-the-pocket-like preventive strategies through HRV monitoring in wearable devices. RMSSD, root mean square of successive differences; PAS, percentage of alternating segments; SODP Q1, points in the first quadrant of the second-order difference plot; SODP CTM100, points within 100 ms radius circle at origin; SD1/SD2, Poincaré plot ratio.

## Introduction

Recent guidelines from the European Society of Cardiology highlight the importance of risk factor management in atrial fibrillation (AF) through the implementation of the newly proposed AF-CARE framework.^1^ These factors can be assessed using clinical scores, genetic risk scores, and machine learning (ML) algorithms, whose performance varies depending on the specific algorithms and databases employed^2–9^ These methods for identifying at-risk patients enable preventive interventions, including dietary modifications and management of comorbidities.^9^ However, they do not offer precise information regarding the timing of AF onset.

Early detection and prediction of the onset of AF could facilitate targeted interventions, potentially preventing or mitigating disease progression and improve patient outcomes. Knowing that an episode of AF can occur within a few hours may lead to a more selective preventive strategy. Using ML with two-lead Holter allows the development of predictive models for paroxysmal AF within such a short window. Implementing an ML model in wearables or connected devices may prove to be an effective prevention tool, ultimately allowing for an optimized pill-in-the-pocket (PITP)-like strategy.

This work focused on identifying patients who are still in sinus rhythm but will develop an AF episode in the coming hours using 24-h Holter recordings and ML.

## Methods

### Dataset construction

We created a new database consisting of Holter recordings from four hospitals and one outpatient clinic. A total of 95 871 recordings were manually visualized. Recordings with AF (irregular RR intervals [i.e., irregular time between successive R-wave peaks] and absence of P wave recorded for at least 30 s) were annotated to serve as ground truth. The Holter recording systems used consisted of two-channel SpiderView digital recorders (Microport CRM, Clamart, France).

#### Patient selection and annotation

**Figure 1:**
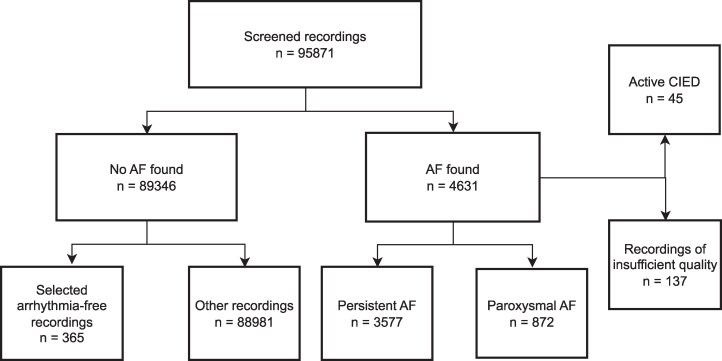
Flowchart of Holter ECG recordings selection.

The inclusion criteria for this study were as follows: adults aged over 35 years with at least one AF event detected by Holter. The exclusion criteria were persistent/permanent and the presence of a cardiac implantable electronic device (CIED) because it is not possible to calculate heart rate variability (HRV) in individuals with electrically paced rhythms. Recordings were transferred from the recorders to the Microport analysis software SyneScope (version 3.30a, Microport CRM, Bagneux, France) for an initial correction to eliminate the coarsest artefacts of the complexes. All recordings were subsequently edited and visually reanalysed in their entirety to search for all AF episodes longer than 30 s. Overall,1319 paroxysmal AF episodes were labelled from 872 patients (*Figure 1*). All recordings were once again reanalysed to determine the exact beginning and end of each paroxysmal AF episode. This allows for precise analysis of the transition from sinus rhythm to AF episodes. Each recording contained both sinus rhythm and one or more AF episodes. This process has been described in detail.^10^

Among these, 835 AF episodes from 506 recordings had more than 60 min of sinus rhythm before AF onset and more than 10 min of AF after onset, and a total of 964 AF episodes had 30 min or more sinus rhythm before AF onset and 5 min or more of AF after onset. Patients with AF episodes lasting more than 30 s, 5 min, or 10 min were considered. The patients were divided into five groups: all patients, patients aged younger than 60 years, 60–70 years, 70–80 years, and older than 80 years.

A total of 365 recordings from 347 patients without rhythm or excitability abnormalities were identified in our database and classified (*Table 1*). To increase the amount of data available for model training and increase the variability of the data for arrhythmia-free subjects, we included the 12th hour, which is the hour in the middle of the recording.

**Table 1:** Number of patients, recordings, and windows according to age groups (with AF recordings > 5 minutes)

Age	Patients			Recordings			1-h windows		
	All	AF	NSR	All	AF	NSR	All	AF	NSR
<60	255	78	177	266	86	180	476	116	360
60–70	238	142	96	252	154	98	397	199	198
70–80	210	145	65	222	155	67	337	199	138
>80	136	116	20	139	119	20	180	140	40

### Machine learning model development

**Figure 2:**
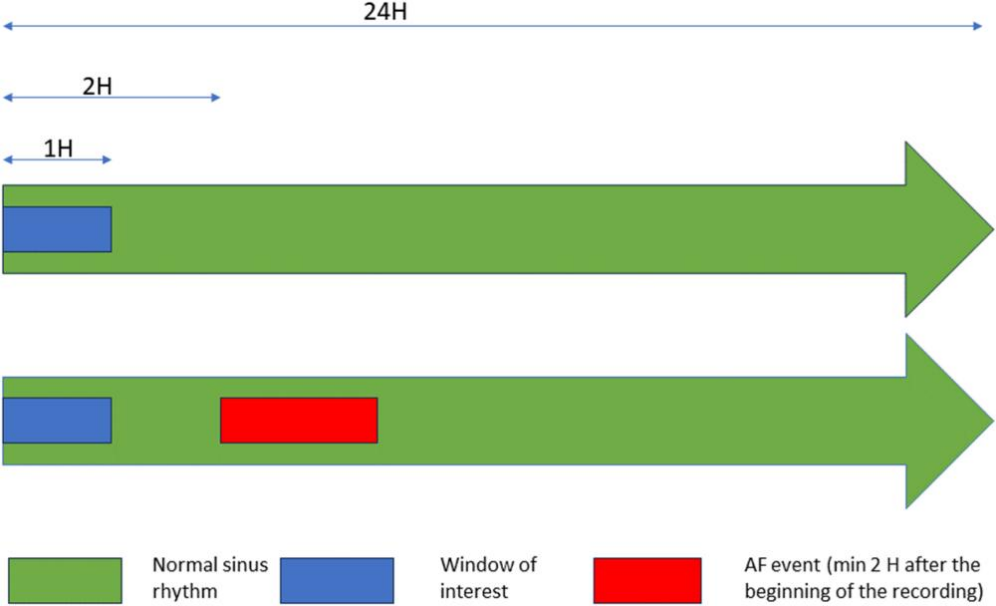
Temporal selection of analysis windows: each arrow represents a 24-h holter recording. One-hour window of interest was selected after the beginning of the recording, either during normal sinus rhythm (top) or preceding an atrial fibrillation event (bottom). Only atrial fibrillation events occurring at least 2 h after the start of the recording were included in the analysis.

Four ML models were used to identify paroxysmal AF patients from the first 30 min of the electrocardiogram (ECG) in normal sinus rhythm (NSR) without signs of AF during the first hour (*Figure 2*). For arrhythmia-free recordings, 30 min of ECGs in the NSR were analysed at the beginning and middle of the recordings.

### Deep learning models

Two deep learning (DL) models were utilized to make predictions from the raw ECG data. The first model was a deep neural network (DNN), which corresponds to the model used in several state-of-the-art studies.^5^ The second model used was a ResNet convolutional neural network (CNN-RNN) using one-dimensional data.^11^

### Decision tree-based models

To compare the performance of our DL models with that of two decision trees using HRV parameters as features, we selected a random forest (RF) classifier and a gradient-boosted decision tree XGBoost (XGB) model. These models used short- and long-term HRV parameters and fragmentation indices as inputs. These were used as follows:

- Sixteen time domain features: mean heart rate, average heart rate in beats per minute (b.p.m.); SDNN [standard deviation of normal-to normal (NN) intervals], standard deviation of all NN intervals; a global measure of HRV; RMSSD (root mean square of successive differences), square root of the mean of the squared differences between successive RR intervals; reflects short-term HRV; SDSD (standard deviation of successive differences), standard deviation of successive RR interval differences; CVNN (coefficient of variation of NN intervals), relative variation of NN intervals (SDNN divided by mean NN interval); CVSD (coefficient of variation of successive differences), relative variation of successive RR interval differences (SDSD divided by mean NN interval); pNN10, pNN20, and pNN50, percentage of successive NN interval pairs differing by more than 10, 20, and 50 ms, respectively; minNN/maxNN, minimum and maximum values of NN intervals; medianNN, median value of NN intervals; prc20NN/prc80NN, 20th and 80th percentiles of the NN interval distribution; TINN (triangular interpolation of NN interval histogram), baseline width of the NN interval histogram; a global HRV index; HRVi (HRV triangular index); and total number of NN intervals divided by the height of the histogram of all NN intervals.
- Five frequency domain features: total power, power low frequency [LF: (0.04–0.15 Hz)] band, power high frequency (HF: (0.15–0.4 Hz) band, normalized values of LF and HF bands, and LF/HF ratio.
- Thirteen nonlinear features: Poincaré plot features, SD1 (standard deviation perpendicular to the line of identity), SD2 (standard deviation along the line of identity), and the SD1/SD2 ratio; the cardiac sympathetic index (CSI); the cardiac vagal index (CVI); and modified CVI. Second-order difference plot (SODP) features: the number of ΔRR in Q1 to Q4 (number of RR interval differences in each of the four quadrants of the SODP), the central tendency measure (CTM)20, the CTM50, and the CTM100 (proportion of SODP points within a circle of radius 20, 50, or 100 ms centred at the origin).
- Five long-term variability features: acceleration (AC), deceleration (DC), AC-modified, DC-modified, Ack (Variant of the acceleration parameter), and dDCk (variant of the deceleration parameter).
- Four parameters were calculated from the heart rate fragmentation (HRF) indices: percentage of inflection points (PIP), inverse of the average length of the acceleration/deceleration segments (IALS), percentage of short segments (PSS), and percentage of alternating segments (PAS).

### Data preparation and input strategy

A single 5-min window was used as input for window-level predictions, and all 5-min windows were averaged for hourly recording-level predictions, allowing us to evaluate performance at two different levels: 5 min or 1 h. Because 1-h windows were too large for direct use by the models, they were divided into overlapping 5-min segments. This approach substantially increased the number of usable data points in the final dataset.

### Cross-validation strategy

The models were evaluated using temporal 10-fold cross-validation at the patient level. The recordings were ordered by their recording date and divided into 10 groups. In turn, each of the 10 groups was used as a test set, and the remaining nine groups were used as a training set, corresponding to a 90–10% training-to-test ratio. If a validation procedure was useful during model training, a validation set was also set aside, and the model was trained on the remaining eight groups, i.e. an 80–10–10% train–validation–test ratio. Note that the separation was performed at the patient level to avoid any data contamination between the training and test sets. If a patient had more than one recording, the first recording was used as the reference date, and all the recordings of that patient were in the same group. If multiple recordings from the same patient were selected, those recordings were assigned to the same split to avoid contamination of the data in the training–testing process.

### Preprocessing

For the correct use of HRV parameters in the decision tree-based models, we used cubic spline interpolation. Premature atrial contractions (PACs) were not taken into account^12^ in decisions trees because inferences about the role of the autonomic nervous system (ANS) only make sense in sinus rhythm.

### Performance evaluation

The area under the receiver operating characteristic curve (AUROC) was calculated, and since the different age groups were not balanced and the incidence of AF varied with age, area under the precision–recall curve (AUPRC) analysis was used to evaluate the ML-based AF prediction models. The sensitivity, specificity, positive predictive value (PPV), negative predictive value (NPV), F1 score, and accuracy were also evaluated to analyse the performance of the models. When the model used windows smaller than 1 h, we first evaluated the window level and then aggregated all the windows for a single recording and evaluated the model at the recording level.

To interpret the contribution of each feature to the predictions made by the decision tree models, we used SHapley Additive exPlanations (SHAP) scores.^13^ Normally, distributed data are presented as the mean ± SD.

### Ethics approval

This study was approved by the ethical committees of Erasme, Brugmann, Ambroise Paré University Hospitals and Europe Hospitals in Belgium and by the Luxembourg National Research Ethics Committee.

## Results

For all patients included, the mean age at the time of Holter monitoring was 64.9 years (± 13.9). *Table 1* shows the results by age group for patients, recordings, and windows. Patients with AF had a mean of 2.3 ± 2.2 (range 1–11) episodes. The CHA_2_DS_2_-VASc score was 2.9 ± 1.7 (range 1–9), which was derived from only two centres, as it was not possible to retrieve all clinical data for the other centres due to the anonymization process. The mean duration of AF events was 3H32 ± 5H58, occurring 15H08 ± 13H05 after the start of recording.

**Figure 3:**
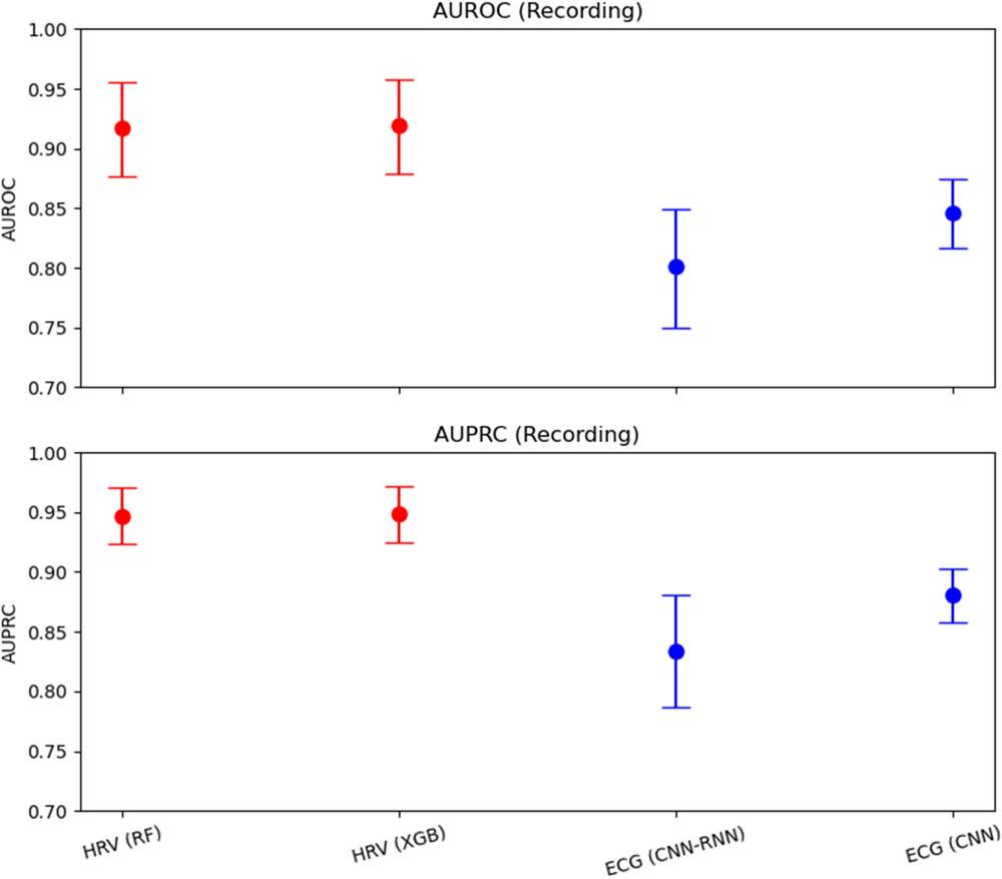
Comparison of model performance for heart rate variability-based (RF and XGB) and ECG-based (CNN-RNN and CNN) models. Mean area under the receiver operating characteristic curve and area under the precision–recall curve values at the recording level are shown with their 95% confidence intervals. Heart rate variability-based models (RF and XGBoost) demonstrate consistently higher performance than ECG-based models (ResNet convolutional neural network and convolutional neural network).

**Figure 4:**
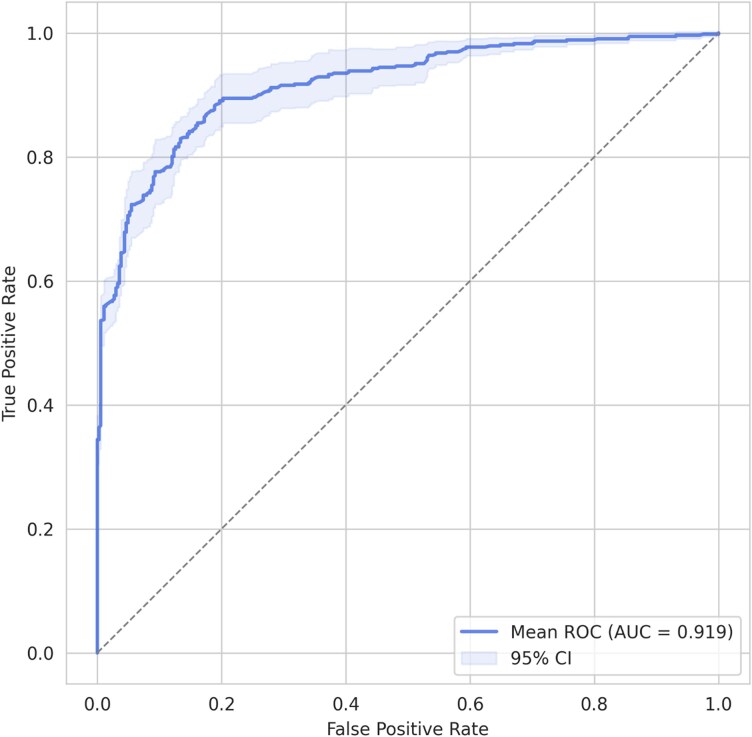
Area under the receiver operating characteristic curve of the XGBoost model (all atrial fibrillation patients; 1-H window; atrial fibrillation duration > 5 min).

**Figure 5:**
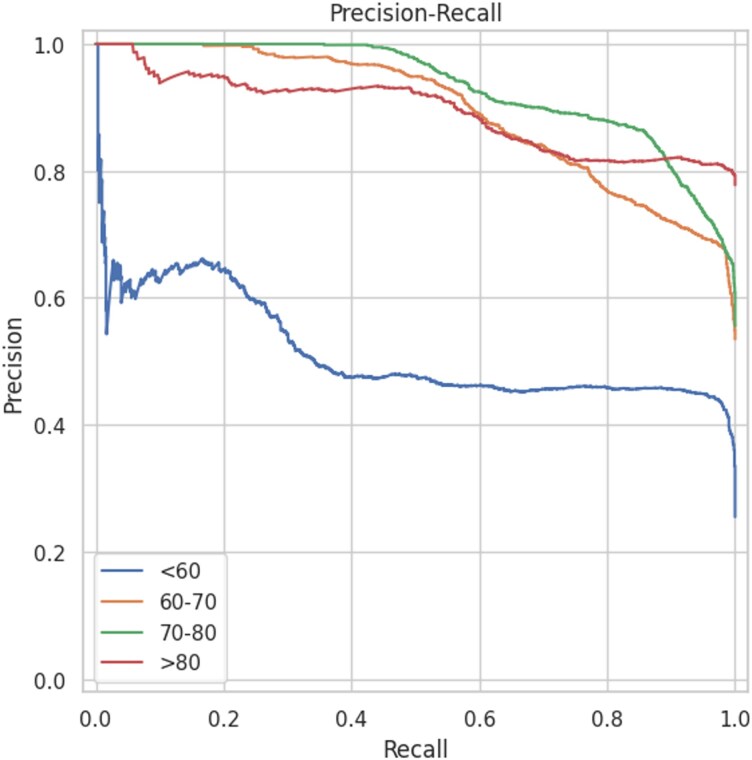
Precision–recall curves for the different age classes and atrial fibrillation durations > 5 min. The area under the precision–recall curve allows for better differentiation between age categories and shows that the algorithms achieve higher performance in age groups above 60 years.

The most significant results were obtained from recordings with more than 5 min of AF episodes (*Table 2*). We compared the performance of the decision tree models with that of the two DL models (*Figure 3*). The best performance was achieved by the models using HRV features. When evaluated at the recording level, the XGB model achieved an AUROC of 0.919 (95% CI: 0.879–0.958) (*Figure 4*) and an AUPRC of 0.919 (0.879–0.958) (*Figure 5*). A threshold of 0.5 was used, which corresponded to an accuracy of 84.5% (81.2–87.8), a sensitivity of 83.0% (79.5–86.4), a specificity of 86.6% (79.3–93.9), a PPV of 90.2% (85.5–94.9), an NPV of 78.4% (74.7–82.1), and an F1 score of 86.2% (83.5–89.0) for the all-patient group. The RF model yielded similar performance metrics.

**Table 2:** Model performances assessed at both the window and recording levels for atrial fibrillation episodes lasting more than 5 min

Input	Model	Window size (seconds)	AUROC (windows)	AUPRC (windows)	AUROC (recording)	AUPRC (recording)
HRV	RF	300	0.866 (0.818–0.913)	0.863 (0.818–0.908)	0.917 (0.877–0.956)	0.947 (0.923–0.971)
HRV	XGB	300	0.864 (0.815–0.913)	0.864 (0.818–0.909)	0.919 (0.879–0.958)	0.949 (0.925–0.972)
ECG	CNN-RNN	30	0.754 (0.701–0.807)	0.696 (0.624–0.769)	0.801 (0.750–0.849)	0.834 (0.787–0.881)
ECG	CNN	30	0.809 (0.780–0.838)	0.760 (0.724–0.796)	0.846 (0.816–0.875)	0.881 (0.858–0.903)

Including age and sex as input variables did not provide any additional benefit in tree-based models (*Table 3*).

**Table 3:** Comparison of model performance using heart rate variability features alone vs. heart rate variability features combined with age and sex, for 300-s analysis windows

Input	Model	Windows AUROC (95% CI)	Windows AUPRC (95% CI)	Recording AUROC (95% CI)	Recording AUPRC (95% CI)
HRV	RF	0.866 (0.818–0.913)	0.863 (0.818–0.908)	0.917 (0.877–0.956)	0.947 (0.923–0.971)
HRV + A + S	RF	0.862 (0.798–0.927)	0.861 (0.824–0.938)	0.918 (0.872–0.964)	0.954 (0.926–0.971)
HRV	XGB	0.864 (0.815–0.913)	0.858 (0.810–0.910)	0.911 (0.879–0.949)	0.949 (0.925–0.972)
HRV + A + S	XGB	0.904 (0.853–0.955)	0.903 (0.848–0.958)	0.950 (0.905–0.981)	0.948 (0.916–0.981)

For all the input sizes, we observed an improvement when the model was evaluated at the recording level rather than at the window level. For both the RF and XGB models, we observed an increase in performance for both the AUROC and AUPRC with longer windows. For the recording-level evaluation, the 5-min window yielded the best results, outperforming the 1-h window (*Table 4*). We observed the same increase in performance between the window evaluation and the recording evaluation for the DL models, but the performance was lower than that of the RF and XGB models. The CNN model performs better than the CNN–RNN model using the same input window.

**Table 4:** Performance of the XGBoost model using heart rate variability parameters computed on 5-min and 1-h windows according to age categories

	Age group	One single window	One single window	Averaged windows	Averaged windows
		AUROC	AUPRC	AUROC	AUPRC
HRV 5 min	<60	0.850 (0.799–0.901)	0.688 (0.588–0.789)	0.904 (0.850–0.959)	0.866 (0.796–0.937)
	60–70	0.844 (0.810–0.877)	0.844 (0.800–0.887)	0.933 (0.891–0.975)	0.957 (0.929–0.985)
	70–80	0.784 (0.720–0.848)	0.848 (0.798–0.898)	0.845 (0.770–0.920)	0.934 (0.901–0.967)
	>80	0.893 (0.857–0.929)	0.965 (0.953–0.977)	0.983 (0.962–1.004)	0.997 (0.994–0.999)
HRV 1 h	<60	0.888 (0.835–0.941)	0.745 (0.646–0.844)	0.898 (0.846–0.949)	0.846 (0.768–0.924)
	60–70	0.877 (0.845–0.909)	0.872 (0.826–0.919)	0.911 (0.869–0.952)	0.948 (0.924–0.972)
	70–80	0.840 (0.778–0.902)	0.878 (0.823–0.932)	0.860 (0.779–0.941)	0.943 (0.908–0.977)
	>80	0.907 (0.861–0.952)	0.970 (0.951–0.990)	0.963 (0.923–1.004)	0.994 (0.988–0.999)

Using the XBG models on HRV features from 5-min ECG windows, we evaluated the performance of the model using the four selected age groups. The best performance was obtained in the >80 group, with an AUROC of 0.983 and an AUPRC of 0.997. The AUPRC was lower for the <60 years group because the prevalence was lower in this group. The accuracy reflects the performance of the AUROC and AUPRC, with a lower performance in the 70–80 years age group. The sensitivity and PPV increase with age, with a 98% sensitivity and 95% PPV for patients aged 80 and older (*Table 5*).

**Table 5:** Metrics of the XGBoost model according to age for an atrial fibrillation duration > 5 min

Age group	Accuracy	Sensitivity	Specificity	PPV	NPV	F1
<60	0.835 (0.772–0.897)	0.582 (0.415–0.749)	0.956 (0.910–1.001)	0.882 (0.764–0.999)	0.834 (0.774–0.895)	0.668 (0.516–0.820)
60–70	0.849 (0.792–0.907)	0.813 (0.729–0.897)	0.906 (0.836–0.975)	0.935 (0.894–0.976)	0.771 (0.679–0.862)	0.866 (0.811–0.920)
70–80	0.770 (0.685–0.855)	0.845 (0.769–0.920)	0.595 (0.417–0.774)	0.833 (0.768–0.899)	0.625 (0.472–0.779)	0.836 (0.775–0.898)
>80	0.935 (0.906–0.964)	0.984 (0.960–1.008)	0.650 (0.409–0.891)	0.946 (0.910–0.982)	0.926 (0.813–1.039)	0.963 (0.947–0.979)

**Figure 6:**
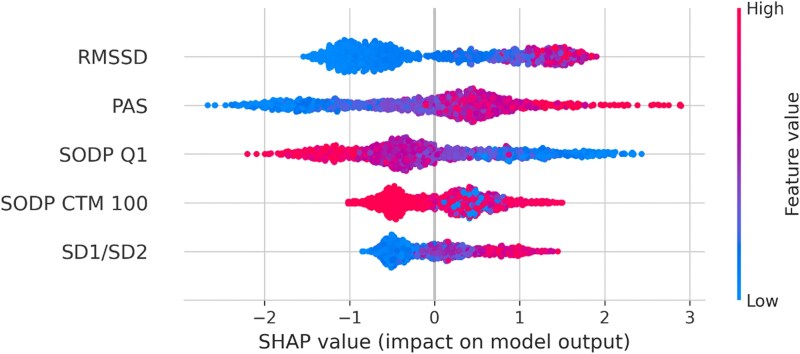
Beeswarm plot of the five most significant SHapley Additive exPlanations values, allowing US to rank the importance of the HRV features used by our XGBoost model. The red dots indicate higher feature values, and the blue dots indicate lower feature values. RMSSD, root mean square of successive differences; PAS, percentage of alternating segments; SODP Q1, points in the first quadrant of the second-order difference plot; SODP CTM100, points within 100 ms radius circle at origin; SD1/SD2, Poincaré plot ratio.

We used a beeswarm plot to summarize the entire distribution of SHAP values for each feature (*Figure 6*). SHapley Additive exPlanations decomposes the output of the model by the sum of the impacts of each feature and allows interpretation of the predictive ML model in search for causal insights. The parameters used by our XGB model were in the order of importance: RMSSD, PAS, SODP Q1, SODP CTM, and SD1/SD2 before the next 45 dependent features. Thus, most of the information derived from the decision tree was contained in the short-term indices of HRV.

## Discussion

Our results indicate that it is possible to identify patients who will develop AF <24 h before event onset. In our retrospective analysis, the PPV was 90.2% overall and increased to 94.6% for patients aged over 80 years, who represent the highest-risk group for developing AF. Notably, our models demonstrated higher sensitivity in older patients and higher specificity in younger patients, which is clinically beneficial. Furthermore, most predictive information was derived from the analysis of HRV. Neither age nor sex was included in our models, confirming that the integration of demographic variables, such as age and sex, into ML models does not provide any significant benefit in terms of predictive performance. This suggests that these variables may be redundant with features already extracted from the ECG signals.^14,15^ To date, there is no evidence that HRV parameters can reliably be used to infer age and sex, as suggested by our tree-based models. However, it is well established that HRV does vary with both age and sex.^16–19^

An important point concerns PACs, whose frequency increases prior to AF^20^ episodes. Although they were included in the input data for the DL models, they were excluded from the data used as input for the decision tree-based models as they distort the calculation of HRV parameters.^12^ Premature atrial contractions are essential triggers for initiating AF episodes, and the increase in their frequency is preceded by modulations of the ANS.^20^ Our results suggest that the decision trees rely on ANS modulations to make their predictions and that the presence of premature beats is not required for accurate prediction.

According to the SHAP score, the parameters used by the decision trees are, in the order of importance, RMSSD, PAS, SODP Q1, SODP CTM, and SD1/SD2. This means that the model identified these parameters as the ones that make the best prediction. Note that many of the parameters entered into the model are correlated; however, the model has made the most appropriate selection, and these first five parameters are uncorrelated or weakly correlated.

The RMSSD parameter ranked by our model is a statistical temporal HRV parameter calculated over 5-min periods and is an indicator of short-term vagal activity.^21^ Thus, XGB indicates vagal activity (for 300 beats) as the primary culprit.^22^ The second parameter ranked by our model is PAS. It is a fragmentation index that calculates the percentage of NN intervals in alternation segments. An alternation segment is a sequence of at least four NN intervals for which heart rate acceleration changes sign every beat. This phenomenon must be distinguished from SODP, as it is more of a high short-term HRV consistent with the breakdown of the neuroautonomic-electrophysiological control system associated with ageing and the onset of cardiovascular disease.^23,24^ This fragmentation parameter is associated with abnormal ANS function, possibly in the context of sick sinus disease, which in turn is associated with AF.^25^ The third-ranked parameter is the first quadrant of the SODP of the interval variation diagram, which is composed of the differences in the RRs compared with the previous differences. This quadrant shows the slowing of the heart rate over three consecutive RR intervals, revealing the very short-term vagal influence.^26^ This highlights the importance of very rapid, very short-term vagal activity, as evidenced by the differences in RR intervals over three beats, whose meaning is different from that of the variations in RR intervals over 300 beats for the RMMSD parameters. The fourth-ranked parameter, the SODP CTM, is a parameter adopted to quantify the degree of variability in an SODP. It is a measure of the total variability of the signal: short-term, long-term, and non-linear components.^27^ This suggests that some non-linear effects may also be at play. The fifth factor is the relationship between the short- and long-term variabilities in RR intervals. The width (SD1) of the Poincaré plot corresponds to the level of short-term HRV, while the length (SD2) of the plot corresponds to the level of long-term variability. Although these plots were originally constructed to measure non-linearities, the SD1/SD2 ratio correlates with linear temporal measures of variability.^27^ All these findings confirm the already known role of the ANS in the initiation of AF, both in normal and dysfunctional states.^20,28,29^ Therefore, short-term HRV indices associated with vagal tone were the most informative predictors in this dataset. Note that 5 min of recording already gives very acceptable predictive performance (*Table 5*), that 5 min accounts for short-term HRV, and that this is all consistent.

Of particular interest is the fact that RR intervals are more relevant when time series are used. In this case, the key information is contained in the timing of the R-waves. Therefore, you do not need to have all 12 leads, and even a single lead contains useful information. Moreover, a photoplethysmography (PPG) could be used to calculate important indices since perfect equivalence can be achieved with the variability calculated from RR intervals or pulse waves.^30^ With the emergence of mHealth techniques, the ability to derive relevant information from a single-lead ECG or a PPG device to make predictions on the order of the day is becoming increasingly appropriate.^31^ A recent study indicated that large-scale screening for AF is feasible using only a smartphone with a dedicated application based on PPG technology.^32^ A ML model for real-time prediction of AF has been developed and validated in a population at very high risk of developing AF using PPG.^33^

Our results offer hope for the development of short-term preventive therapeutic strategies in which patients can be alerted to take medication before an AF crisis based on the predicted onset of an AF episode. A nasal spray has recently been shown to be effective and could be prescribed for this purpose in view of the need for fast action.^34^ In this case, the patient would take their medication while still in sinus rhythm. This approach could help prevent the infrequent instances of adverse reactions associated with the current PITP strategy, which is implemented when the patient is already in AF.^35^ The PITP strategy refers to the use of a Class 1c antiarrhythmic drug when the patient experiences palpitations but is not systematically used.^36^ Nevertheless, a PITP-like strategy could be considered for patients still in sinus rhythm. A potential application could involve the use of a connected watch or wearable device that can alert the patient when a signal indicating an incoming AF episode is received. In such cases, the patient can be prompted to take appropriate medication to prevent an AF episode. In the current context, there is no clinical proof of this concept, and this justifies the need for studies to prospectively validate these initial results and confirm that an ML-PITP-like strategy based on ANS modulations is feasible for short-term AF prevention using our model. This strategy warrants further discussion; however, combining a fast-acting flecainide nasal spray with oral propafenone could represent a promising approach to optimize both rapid onset and sustained antiarrhythmic effects. In this case, the goal is to reduce the AF burden in patients who are already receiving anticoagulant therapy in the hope of slowing the progression to long-term permanent AF. However, although the models showed promising results on the test set, the lack of rigorous internal or external validation means that their generalizability to other datasets or clinical settings remains uncertain. We would like to emphasize that the use of such ML models still needs to be established and prospectively confirmed.

### Study limitations

This was a retrospective study based primarily on Holter data from mixed patients, some of whom were receiving antiarrhythmic therapy. This introduces relative heterogeneity into our sample, but on the other hand, it is more in line with the real clinical situation that our model will have to address. Although we were particularly careful in the selection of patients considered normal and without arrhythmia, the Holter recordings labelled as arrhythmia-free could have had other pathologies because clinical characteristics were not available for all subjects, introducing some bias. However, it is likely that the results would have been even better if healthy volunteers had been recruited for the study. In fact, it is not easy to obtain arrhythmia-free data retrospectively, especially for individuals older than 80 years. In addition, to balance the database of patients with AF and patients without AF, we selected two 1-h windows from among subjects without AF. This could also introduce bias into the analysis. Moreover, the reported performance metrics are based solely on the test set, with neither proper internal nor external validation performed, an important limitation to consider when interpreting our results. Although this is the largest Holter database regarding paroxysmal AF episodes recorded, the overall number of episodes remains limited. Additionally, only the first hour of each paroxysmal AF recording and two one-hour segments of NSR were included. To make the data suitable for the models, these 1-h windows were divided into overlapping 5-min segments, thereby increasing the number of usable data points.

## Conclusions

Machine learning techniques using Holter ECG recordings can identify patients who develop AF episodes a few hours before they occur. The best results were achieved with our decision tree-based models. A comparison of the results obtained by two different ML techniques, namely interpretable decision trees and the *a posteriori* explicable DNNs, suggested that the important information enabling the prediction of AF onset is contained in HRV. This finding reinforces the role of ANS modulation in the initiation of AF episodes and that HRV can be used to make the predictions that allow a preventive strategy. This opens up perspectives that can be exploited by wearables in mHealth, and in this context, the use of a PITP-like preventive strategy to reduce the burden of AF. Prospective studies are needed to confirm the encouraging potential of these findings.

## Data Availability

The first part of the database (IRIDIAv1) can be downloaded from Zenodo: Cédric Gilon, Jean-Marie Grégoire, Marianne Mathieu, Stéphane Carlier, & Hugues Bersini (2023). IRIDIA-AF, a large paroxysmal atrial fibrillation long-term electrocardiogram monitoring database (1.0.1) [Data set]. Zenodo. https://doi.org/10.5281/zenodo.8405941. IRIDIA_v2 (the expanded database used in this work) can be obtained from the first author upon reasonable request
